# In Vitro Analysis of the Effect of SARS-CoV-2 Non-VOC and four Variants of Concern on MHC-Class-I Expression on Calu-3 and Caco-2 Cells †

**DOI:** 10.3390/genes14071348

**Published:** 2023-06-26

**Authors:** Nora A. Bahlmann, Lena Mautner, Mona Hoyos, Erwan Sallard, Carola Berger, Alexandra Dangel, Franziska Jönsson, Johannes C. Fischer, Florian Kreppel, Wenli Zhang, Irene Esposito, Edwin Bölke, Armin Baiker, Anja Ehrhardt

**Affiliations:** 1 Virology and Microbiology, Center for Biomedical Education and Research (ZBAF), Witten/Herdecke University, 58453 Witten, Germany; 2 Bavarian Health and Food Safety Authority, 85764 Oberschleißheim, Germany; 3 Biochemistry and Molecular Medicine, Center for Biomedical Education and Research (ZBAF), Witten/Herdecke University, 58453 Witten, Germany; 4 Institute for Transplant Diagnostics and Cell Therapeutics, Heinrich-Heine-University, 40204 Duesseldorf, Germany; 5 Institute of Pathology, Heinrich Heine University and University Hospital, 40204 Duesseldorf, Germany; 6 Department of Radiation Oncology, Heinrich-Heine-University, 40204 Duesseldorf, Germany

**Keywords:** SARS-CoV-2 variants, MHC-I, HLA, Variants of Concern

## Abstract

As the MHC-I-pathway is key to antigen presentation to cytotoxic T-cells and, therefore, recognition by the host adaptive immune system, we hypothesized that SARS-CoV-2 including its Variants of Concern (VOCs), influences MHC-I expression on epithelial cell surfaces as an immune evasion strategy. We conducted an in vitro time course experiment with the human airway epithelial cell line Calu-3 and the human colorectal adenocarcinoma cell line Caco-2. Cells were infected with SARS-CoV-2 strains non-VOC/B.1.1, Alpha/B.1.1.7, Beta/B.1.351, Gamma/P.1, and Delta/B.1.617.2. At 2, 24, 48 and 72 h post-infection we performed RT-qPCR to track viral replication. Simultaneously, we performed intracellular staining with a serum of a double-vaccinated healthy adult containing a high amount of spike protein antibody. In flow cytometry experiments, we differentiated between infected (spike protein positive) and bystander (spike protein negative) cells. To compare their HLA expression levels, cells were stained extracellularly with anti-HLA-A-IgG and anti-HLA-B,C-IgG. While HLA-A expression was stable on infected Calu-3 cells for all variants, it increased to different degrees on bystander cells in samples infected with VOCs Beta, Gamma, Delta, or non-VOC over the time course analyzed. In contrast, HLA-A levels were stable in bystander Calu-3 cells in samples infected with the Alpha variant. The upregulation of MHC-I on spike protein negative bystander cells in Calu-3 cell cultures infected with Beta, Gamma, Delta, and partly non-VOC might suggest that infected cells are still capable of secreting inflammatory cytokines like type-I interferons stimulating the MHC-I expression on bystander cells. In comparison, there was no distinct effect on HLA expression level on Caco-2 cells of any of the VOCs or non-VOC. Further investigations of the full range of immune evasion strategies of SARS-CoV-2 variants are warranted.

## 1. Introduction

Severe acute respiratory syndrome coronavirus type 2 (SARS-CoV-2) belongs to the betacoronavirus subgroup B. This new betacoronavirus strain emerged in December 2019 in Wuhan, China, as the cause of coronavirus disease 2019 (COVID-19). Symptoms of this disease range from mild to severe and mainly affect the respiratory system, but the virus is also capable of causing gastrointestinal, neurological, and cardiovascular disorders. The enveloped single-stranded RNA virus has caused a global pandemic with over 767 million confirmed cases and more than 6.9 million deaths reported globally until 11 June 2023 [1]. Despite the proofreading ability of the SARS-CoV-2 replication machinery, the virus genome has acquired various mutations during its worldwide spread [2]. The original strain found in Wuhan was rapidly replaced by the D614G mutated variant. Since then, several new variants emerged. These variants have proven to possess a significant impact on transmissibility, virulence, or change in clinical disease presentation and are thereby likely to have an impact on the epidemiological situation [3]. The WHO and national health authorities closely monitor these variants and define them as Variants of Concern (VOCs). The first VOC Alpha/B1.1.7 was identified in the United Kingdom in 2020 and showed higher transmissibility than both former SARS-CoV-2 strains [4,5]. The Alpha was quickly followed by the VOCs Beta/B.1.351 and Gamma/P.1, initially identified in South Africa and Brazil, respectively. These variants had acquired certain immune evasion characteristics, threatening the success of global vaccination efforts [6]. From its detection in April 2021 in India, these variants were rapidly replaced by the VOC Delta/B.1.617.2 [7]. The Delta variant shows increased fitness along with a moderate immune escape, mainly causing its ubiquitous spread [8,9]. In November 2021, Omicron/B.1.1.529 was first reported in South Africa. To date, the highly infectious [10] strain has been the only still circulating VOC, according to the WHO [11]. However, for technical reasons, it could not be included in this study.

The Major Histocompatibility Complex (MHC)-I pathway is key to antigen presentation to cytotoxic T-cells and, therefore, pathogen recognition by the host-adaptive immune system. A number of studies showed that SARS-CoV-2 is able to inhibit MHC-I expression on certain epithelial cells’ plasma membranes as an immune evasion strategy and identified different ORFs responsible for inhibiting MHC-I surface expression [12,13,14]. However, to the best of our knowledge, a direct comparison of several VOCs on their effect on MHC-I expression is lacking. 

Here, we conducted an in vitro analysis with a human lung (Calu-3) and colon cell line (Caco-2). We infected them with the clinical SARS-CoV-2 strains non-VOC/B.1.1, Alpha/B.1.1.7, Beta/B.1.351, Gamma/P.1, and Delta/B.1.617. MHC-I expression on infected Calu-3 cells remained rather stable regardless of the virus variant. However, infection with all virus variants except Alpha increased HLA-A expression on bystander Calu-3 cells to different degrees. In contrast, we found no distinct effect on HLA expression level on Caco-2 cells of any of the VOCs or non-VOC.

## 2. Results

In the present work, we addressed the question of to which extent the different VOCs influence MHC-I expression. Beforehand we had observed a trend towards increased MHC-I expression associated with low numbers of SARS-CoV-2 positive cells (not shown) in lung tissue samples of deceased COVID-19 patients (Supplementary Table S1). Here, we conducted an in vitro analysis using Calu-3 and Caco-2 cells to address whether MHC-I expression changes in SARS-CoV-2 infected and non-infected bystander cells. 

First, we established a protocol to differentiate between spike protein-positive cells and cells with an undetectable level of spike protein that did not support effective virus replication, which is referred to as spike protein-negative bystander cells. As a marker, we used spike protein antibodies containing a serum of a double-vaccinated healthy male adult obtained 20 days after the second vaccination with the mRNA vaccine Comirnaty. The serum was tested in an ELISA beforehand to demonstrate a titer of 1:64,000 spike protein-specific antibodies (Supplementary Figure S1). Subsequently, we used this serum for intracellular staining and combined it with extracellular staining with anti-HLA-A-IgG or anti-HLA-B,C-IgG. Exemplary FACS dot plots of spike protein- and HLA-staining results on Calu-3 are displayed in Figure S2. 

**Figure 1:**
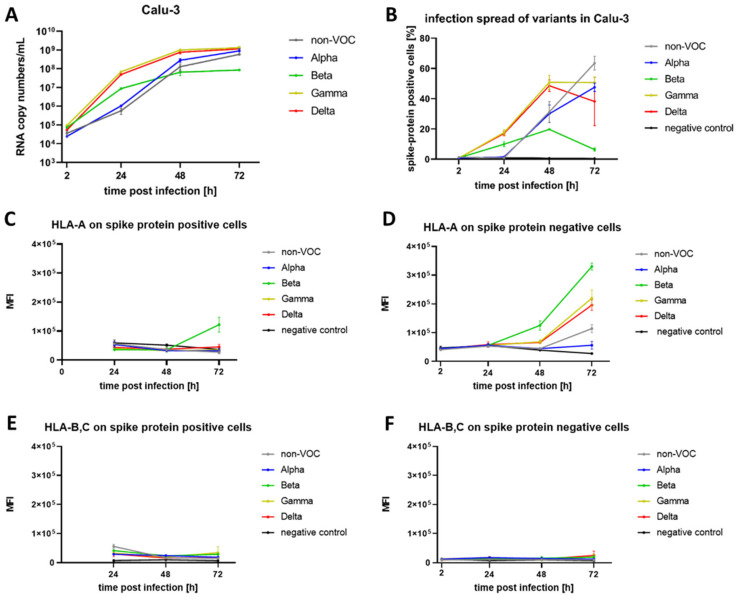
Time course of spike protein, HLA-A, and HLA-B,C expression levels after infection of Calu-3 cells with SARS-CoV-2 strains non-VOC, Alpha, Beta, Gamma, and Delta. Calu-3 cells were infected with the different virus strains at MOI 0.01. Non-infected samples with identical treatment served as a negative control. At indicated time points, cells were analyzed. MFI: Mean Fluorescence Intensity. (**A**) SARS-CoV-2 specific RT-qPCR analysis of the supernatant to track viral replication. (**B**) Spike protein-specific flow cytometry analysis to investigate infection spread of virus strains in Calu-3 cells. Please note that all strains display a strong CPE in Calu-3 cells at later time points, and detached cells were not included in the analysis. (**C**) MFI of HLA-A expression on the surface of spike protein-positive (infected) and (**D**) on spike protein-negative (bystander) Calu-3-cells. (**E**) MFI of HLA-B,C expression levels on spike protein-positive and (**F**) on spike protein-negative Calu-3 cells. The HLA-B,C level remains low over time without a perceivable difference to the negative control. Error bars represent standard deviation (*n* = 3).

We infected Calu-3 cells with the SARS-CoV-2 strains non-VOC, Alpha, Beta, Gamma, and Delta with a multiplicity of infection (MOI) of 0.01. At time points 2, 24, 48, and 72 h, we performed RT-qPCR (Figure 1A) of the supernatant to track viral replication efficacy and collected cells for antibody staining. Note that another time point was planned at 96 h, but due to the strong cytopathic effect (CPE) at this point, cells could not be used for further analysis. While all virus strains replicated efficiently in Calu-3 cells, Alpha, Gamma, and Delta amplified fastest, showing a more than 10,000-fold increase of RNA copy numbers/mL in the first 48 h. In contrast, the fold increase for Beta remained below 1000 RNA copy numbers/mL in this timeframe. Correspondingly, Beta showed the weakest infection spread in Calu-3 cells, determined by measuring spike protein-positive cells in flow cytometry analysis (Figure 1B). 

Simultaneously, we measured HLA-A expression levels on the surface of Calu-3 cells in spike protein-positive cells and spike protein-negative bystander cells. For spike protein-positive cells, we observed that the mean fluorescence intensity (MFI) of HLA-A decreased by 40 % over the 72 h time period, which was similar to the uninfected negative control (Figure 1C). In contrast, in spike protein-negative cells, we detected a strong increase of HLA-A, starting at 48 h (Figure 1D). The effect was most prominent in samples infected with Beta, Gamma, and Delta, showing a more than four-fold increase of MFI over 72 h. In the samples infected with Alpha, the MFI remained stable over time in spike protein-negative cells, while cells infected with non-VOC displayed an intermediate phenotype with a two-fold MFI increase over 72 h. There was no obvious correlation between the virus replication efficacy and the HLA-A expression levels, as Alpha was replicating and spreading comparably well to other variants while there was no rise in HLA-A detectable. In contrast, the weakly replicating strain Beta provoked the strongest increase in HLA-A. Staining with an HLA-A,B,C antibody showed confirmative results (Figure S3), and a preliminary set of data supported the experiment as well (Figure S4). There was no comparable dynamic in surface expression levels of HLA-B,C on spike protein-positive and negative Calu-3 cells detectable (Figure 1E,F). Here, the MFI remained at a constantly low level, without the traceable effect of virus infection. 

The same experiment was also conducted on Caco-2 cells, which are also permissive to SARS-CoV-2 infection. In these cells, all virus variants displayed a weaker replication rate than in Calu-3 cells, even over a time period of 96 h (Figure S5A). Importantly, there was no CPE visible in this timeframe. We detected a constant decline of HLA-A surface expression on spike protein-positive cells similar to the negative control (Figure S5B). On bystander Caco-2 cells, we also observed this decline in HLA-A surface expression. However, the variation between the analyzed virus strains in HLA-A surface expression levels was more evident for bystander cells (Figure S5C). At 96 h, cells infected with Beta showed a 60% higher MFI than cells infected with Alpha and the negative control. There was no variation for HLA-B,C, which remained similar to the control for all virus strains throughout the experiment (Figure S5D,E).

**Figure 2:**
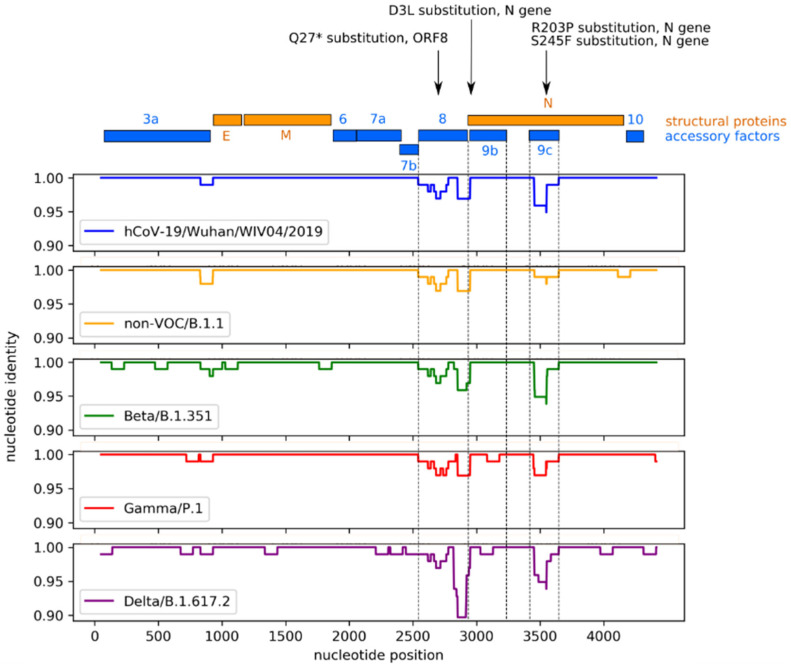
Similarity plot based on SARS-CoV-2 VOCs and non-VOCs. The Alpha (B.1.1.7) was used as a reference sequence. EPI_ISL_402124 (hCoV-19/Wuhan/WIV04/2019): sequence of one of the original Wuhan strains, isolated on 30 December 2019, was included as a comparison in the plot. A multiple alignment of the entire genomic sequence of all variants was conducted using multalin. We then plotted the identity of each variant with Alpha in a 100 nt wide sliding window for each nucleotide position between the 5′ end of ORF3a and the 3′ end of the genome, using Python’s Matplotlib library. We found mutations specific to the Alpha variant in the ORF8 (Q27* non-sense substitution) and ORF9c.

In order to investigate why the infection with Alpha showed a stable MHC-I expression level on bystander Calu-3 cells differing from the other investigated viral strains, we compared its genomic sequence to those of other VOCs and the non-VOC (Figure 2). We found mutations specific to the Alpha variant in the ORF8 (Q27* non-sense substitution) and ORF9c (the R203P mutation in the N gene corresponds to an E51Q substitution). ORF8 is thought to impair type I interferon signaling [15]. It may be assessed in future studies whether the ORF8 truncation or ORF9c mutation is responsible for the suspected immune evasion phenotype of the Alpha variant.

## 3. Discussion

Here we used an epithelial cell culture model to compare the MHC-I surface expression development of four SARS-CoV-2-VOCs Alpha, Beta, Gamma, and Delta and one non-VOC variant. To the best of our knowledge, this is the first published 72 h time course comparison of these variants infecting naturally permissive Calu-3 cells with a distinction between spike protein-positive and negative cells and their MHC-I development. Potentially finding differences between these strains in their escape from cytotoxic T-cell recognition might help targeted therapy against the variants and may even allow a quicker assessment of newly occurring variants. 

Infection with these strains did not lead to an increase in MHC-I surface expression levels on spike protein-positive Calu-3 cells. This matches the fact that several ORFs have been identified to directly or indirectly affect MHC-I surface expression. For instance, ORF6 inhibits interferon-mediated intracellular signaling by targeting STAT1-IRF1-NLRC5, representing important transcriptional regulators of MHC-I [12]. ORF7a inhibits the assembly of the MHC-I peptide loading complex trapping MHC-I in the endoplasmic reticulum [13]. 

The upregulation of MHC-I on spike protein-negative bystander cells in cultures infected with Beta, Gamma, Delta, and partly non-VOC might suggest that infected cells are still capable of secreting inflammatory cytokines like type-I interferons stimulating the MHC-I expression on bystander cells. A recent study revealed that MHC-I expression was upregulated exclusively on bystander cells after rotavirus infection [16]. We found this upregulation only for Calu-3 cells and only for HLA-A surface expression, not B,C. This might be due to cell-specific factors or a difference in promoter activity on the genomic level [17]. Our finding was not reported by other studies analyzing SARS-CoV-2’s influence on MHC-I [12,13,14]. Possible reasons are that these studies did not include Beta, Gamma, and Delta strains for which we observed major MHC-I upregulations and covered shorter time frames. Furthermore, two of these studies did not differentiate between infected and non-infected bystander cells [12,14].

For the Alpha variant, we detected a stable MHC-I expression level on bystander cells and therefore analyzed the major sequence differences of open reading frames coding for accessory proteins of SARS-CoV-2 between Alpha and the other virus strains. The Alpha is the only one of the virus strains compared here with an ORF8 Q27stop mutation, which results in a predicted truncation of the protein by nearly 80% of its length. ORF8 protein has already been associated with mechanisms of immune escape by SARS-CoV-2. It has been described to bind directly to MHC-I in the endoplasmatic reticulum and induce its lysosomal degradation [14]. Taking into account the diverse and partly redundant other means of SARS-CoV-2 to suppress MHC-I expression, it is possible that Alpha compensates for the likely loss of ORF8 protein by overexpressing other ORFs. For instance, Alpha has been shown to express increased subgenomic RNA and protein levels of ORF6 [18]. However, the observed effects of SARS-CoV-2 strains on MHC-I regulation on Calu-3 cells in this purely descriptive work need to be confirmed in further studies, including investigations of the underlying mechanisms. In addition, the cytotoxic activity of T-lymphocytes, depending on the degree of MHC expression inhibition in infected epithelial and bystander cells, could be investigated in future studies.

## 4. Materials and Methods

### 4.1. Cell Culture

The two human cell culture cell lines, Caco-2 (human colorectal adenocarcinoma cells) and Calu-3 (human airway epithelial cells), were cultured in an EMEM growth medium. The medium was supplemented with 20% or 10% fetal calf serum (FCS; Gibco, Invitrogen, Carlsbad, CA, USA), respectively. Also, 1% penicillin–streptomycin (10,000 U/mL, Gibco) was added to the culture medium. Cell culture was maintained at 37 °C and 5% CO_2_ in a humified culture atmosphere. The cells used were kindly donated by Dr. Ulrich Lächelt from the Ludwig–Maximilians University Munich Department of Pharmacy and authenticated by SNP typing. Mycoplasma contamination was excluded as screened by PCR testing.

### 4.2. Isolation and Propagation of Clinical SARS-CoV-2 Strains

Non-VOC/B.1.1, Alpha/B.1.7, Beta/B.1.351, Gamma/P.1, and Delta/B.1.617.2 variants of SARS-CoV-2 were isolated from clinical samples of confirmed SARS-CoV-2 infection as previously described (20). Briefly, swab samples of PCR-positive patients were filtered through a 0.45 µm Minisart syringe (Sartorius Stedim Biotech, Goettingen, Germany) before inoculation on cell culture. When typical cytopathic effect (CPE) was detectable, RT-qPCR (XPRSARS-COV2_10, Cepheid, Sunnyvale, CA, USA) verified the integrity of the isolate. From verified SARS-CoV-2 culture supernatants, viral stocks were generated and stored at −80 °C. For isolation and propagation of SARS-CoV-2 variants Vero E6 (ATCC CRL-1586) were used. Clinical samples, as well as continuous samples from virus propagation, were sequenced by whole-genome sequencing, which confirmed strain identities (see gisaid database (www.gisaid.org accessed on 5 February 2023) and “availability of data and material” for accession numbers). End-point dilution assay determining 50% tissue culture infectious dose (TCID50) was performed on Vero E6 for viral stock titration. Following the applicable security and safety protocols, all experiments containing infectious SARS-CoV-2 virus were performed in a biosafety level 3 laboratory.

### 4.3. SARS-CoV-2 Cell Culture Infection Experiments

One to three days before the infection, Caco-2 and Calu-3 cells were seeded in T-25 cell culture flasks. Cells of one representative T-25 culture flask per cell line were counted after trypsin/EDTA detachment of cells before the infection. Cell lines were infected with the SARS-CoV-2 isolates non-VOC/B.1.1, Alpha/B.1.1.7, Beta/B.1.351, Gamma/P.1, and Delta/B.1.617.2 in triplicates for each analysis time point at an MOI of 0.01 in 1.5 mL of their respective culture media for 2 h. Three samples served as uninfected, negative controls and were handled identically. After washing cells with PBS twice, 4.5 mL of their respective culture media was added. At the time points 2 h (directly after reconstitution in cell culture media), 24, 48, 72, and 96 h post-infection (96 h only for Caco-2), samples were taken from the supernatants of the triplicates for each viral strain for RT-qPCR analysis determining RNA copy numbers. Also, triplicate T-25 flasks of infected cells with each viral strain were fixed for FACS analysis. For observation of the development of CPE, microscopic pictures were taken simultaneously.

### 4.4. Analysis of Extracellular Viral RNA by RT-qPCR

RT-qPCR analysis was performed for the quantification of extracellular viral RNA at each time point. At 2, 24, 28, 72, and 96 h post-infection, supernatant samples were heat-inactivated at 65 °C for 90 min in a heating block. RNA extraction was performed in Maxwell 48 using RSC Blood DNA Kit (AS1400, Promega GmbH, Mannheim, Germany) before RT-qPCR (FTD-114-96, Fast Track Diagnostics, Eschsur-Alzette, Luxembourg) was carried out in a QuantStudio 7 real-time thermal cycler (Thermo Fisher Scientific, Waltham, MA, USA) due to the manufacturer`s instructions. For data acquisition and analysis, the respective QuantStudio Real-Time PCR Software was used. Using purified SARS-CoV-2 RNA, a standard curve was performed for quantification efforts along with each sample measurement. Genome copy numbers of standard curve samples were assessed by RT-ddPCR (reverse transcriptase droplet digital PCR), as previously described [19]. 

### 4.5. Flow Cytometry Preparation

The supernatant was discarded, and cells were washed in PBS before being detached with trypsin/EDTA and transferred into a Falcon tube. After centrifugation, cells were washed twice in PBS before fixation in 200 µL 2% formalin for 30 min at room temperature. Fixed cells were centrifuged, the supernatant was discarded, and cells were washed and resuspended in PBS containing 5% FBS. Samples were stored at 4 °C until further usage.

Cells were treated with an Fc-receptor-blocker (Biolegend, Cat 422302), washed in PBS with 5% FBS and incubated with primary antibodies. The monoclonal antibodies that recognize different HLA proteins were developed and kindly provided by Dr. Soldano Ferrone (Department of Immunology, Roswell Park Cancer Institute, Buffalo, NY, USA). They are LGIII-147.4.1 for HLA-A antigen, B1.23.2 for β2m-free and β2m-associated HLA-B and -C heavy chains, and TP25.99.8.4 for HLA-A,B,C.

After washing, cells were permeabilized and incubated with a serum of a double-vaccinated healthy adult 20 days after full vaccination with Comirnaty. The serum was tested in ELISA to prove that it contains a high amount of spike protein antibody; see below. The serum was heat inactivated at 56 °C for 30 min before use. It was used after optimization in a dilution of 1:150.

After washing, cells were incubated with secondary antibodies (anti-mouse-IgG, Alexa Fluor 488, Cat A21121 Thermo Fisher, Waltham, MA, USA and anti-human-IgG, Dylight 650, Cat SA5-10137, Thermo Fisher), washed again, and analyzed in flow cytometry (Beckmann Coulter, Krefeld, Germany).

### 4.6. ELISA Spike Protein 

To determine the relative anti-SARS-CoV-2 end-point titers, conventional direct ELISA was used. In brief, the S1 domain of SARS-CoV-2 spike protein was expressed in a secreted form in A549 cells (ATCC CCL-185) and purified by affinity chromatography (details can be obtained upon request). The 75ng purified S1 protein was coated per well in a 96-well ELISA plate overnight at pH 8.5. Sera with an initial dilution of 1:2000 was used as the primary antibody, and HRP-conjugated goat anti-human IgG (Abcam, Cambridge, UK, ab97225) was used as a secondary antibody. The titer was determined after cutoff calculation [20] and found to be 1:64,000.

### 4.7. Immunohistochemistry

Autoptic lung tissue samples from three patients deceased of COVID-19-related acute respiratory distress syndrome (ARDS) and one patient deceased from COVID-19 independent causes not affecting either the lungs or the heart were subjected to immunohistochemistry. The MHC-I specific primary antibodies HC-10- HLA class I heavy chain (1:200) and NAMB-1 ß2-microglobulin (1:400) were applied according to the indirect streptavidin–biotin complex method using 3-3′ diaminobenzidine (DAB) as a chromogen. Human tonsil tissue was used as a positive control. Expression was assessed semi-quantitatively in different cell compartments.

## Data Availability

GISAID accession numbers: non-VOC (EPI_ISL_10201358). Alpha (EPI_ISL_10201380), Beta (EPI_ISL_10201351), Gamma (EPI_ISL_10201364), Delta (EPI_ISL_11890620).

## Supplementary Materials

The following supporting information can be downloaded at: https://www.mdpi.com/article/10.3390/genes14071348/s1, Figure S1: anti-spike S1 ELISA; Figure S2: Flow cytometry analysis of spike protein and HLA-A expression levels after infection of Calu-3 cells with five SARS-CoV-2 strains; Figure S3: Time course of spike protein, HLA-A and HLA-B,C expression levels after infection of Calu-3 cells with five SARS-CoV-2 strains; Figure S4: HLA-A,B,C expression levels after infection of Calu-3 cells with SARS-CoV-2 strains non-VOC, Alpha, Beta, Gamma, and Delta, preliminary experiment; Figure S5: Time course of spike protein, HLA-A and HLA-B,C expression levels after infection of Caco-2 cells with SARS-CoV-2 strains; Table S1: HLA-1 staining in lung tissue samples of 3 SARS-CoV2 deceased patients.
