# Supplementary material for: In Vitro Analysis of the Effect of SARS-CoV-2 Non-VOC and four Variants of Concern on MHC-Class-I Expression on Calu-3 and Caco-2 Cells
Source: Genes (Basel). 2023 Jun 26;14(7):1348. doi: 10.3390/genes14071348 (PMC10378856; doi:10.3390/genes14071348)
Supplement: Supplementary file 1 [file genes-14-01348-s001.zip › genes-2338622-supplementary.pdf]

Supplementary Material

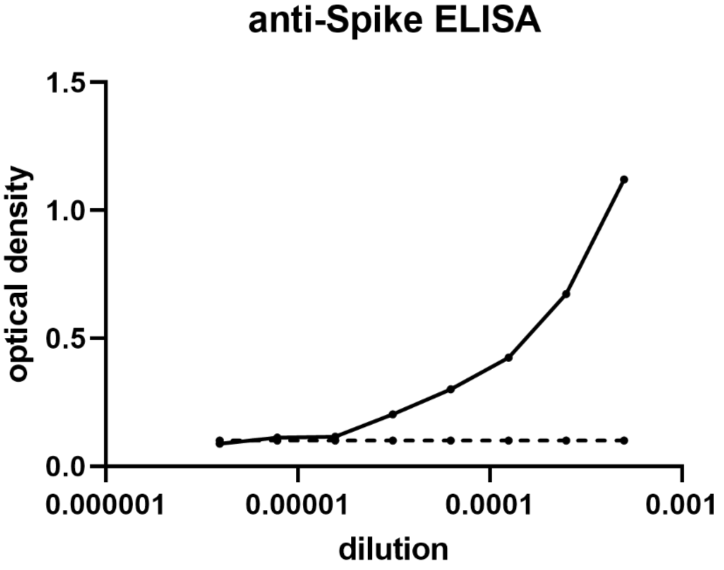

Supplementary Figure S1: anti-spike S1 ELISA. Titer was determined after cutoff calculation and was found to be 1:64,000.

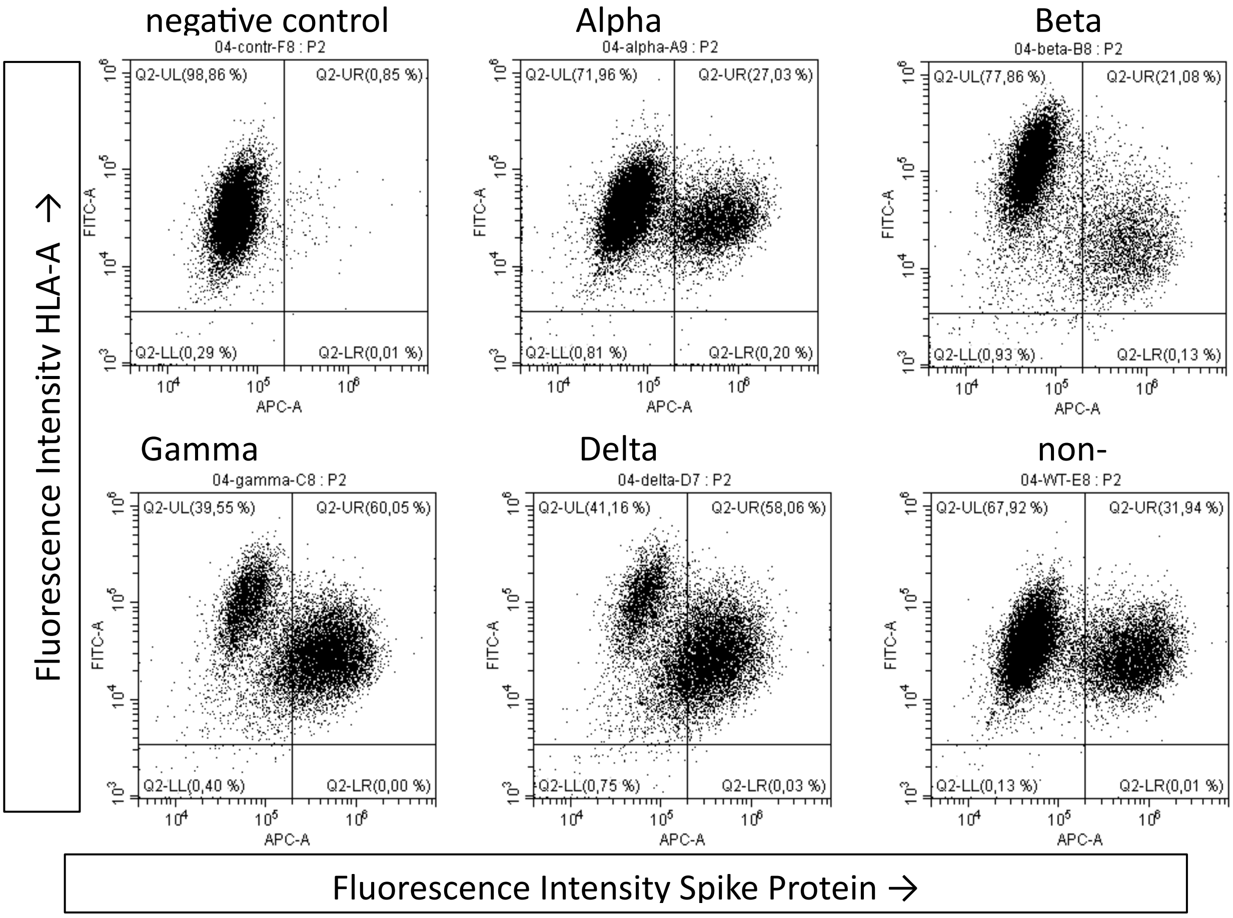

Figure S2: Flow cytometry analysis of spike protein and HLA-A expression levels after infection of Calu-3 cells with SARS-CoV-2 strains non-VOC, Alpha, Beta, Gamma, and Delta. The x-axis represents the level of spike protein in the cells and the y-axis shows the level of HLA-A-expression. Calu-3 cells were infected with the five virus strains using a MOI of 0.01 and 48h post-infection flow

cytometry analysis was performed. Non-infected samples with identical treatment served as negative control. Exemplary dot plots of flow cytometry analysis are shown.

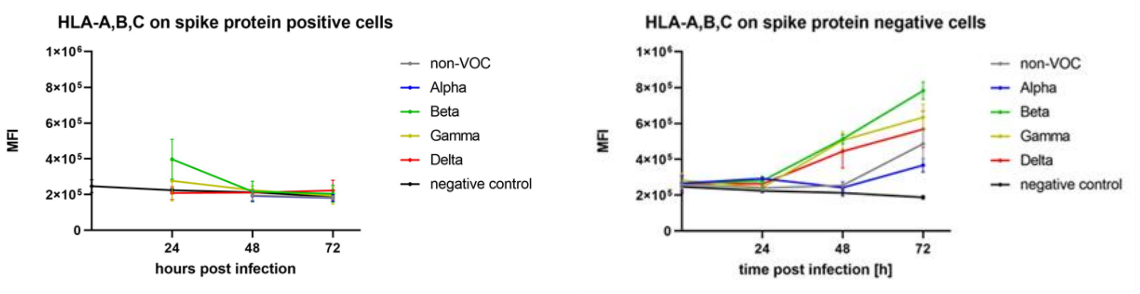

(**A**) (**B**)

Figure S3. Time course of spike protein, HLA-A and HLA-B,C expression levels after infection of Calu-3 cells with SARS-CoV-2 strains non-VOC, Alpha, Beta, Gamma, and Delta. Calu-3 cells were infected with the different virus strains at MOI 0.01. At indicated time points cells were analyzed. Non-infected samples with identical treatment served as negative control. MFI: Mean Fluorescence Intensity. (**A**) MFI of HLA-ABC-expression on the surface of spike protein-positive (infected) and (**B**) on spike protein-negative (bystander) Calu-3 cells. Error bars represent standard deviation (n=3).

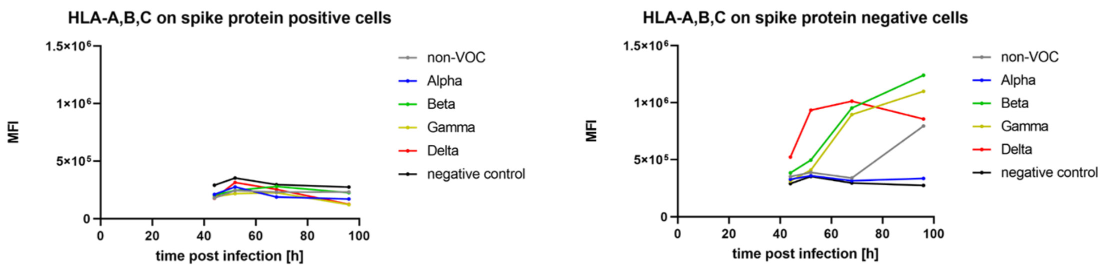

(**A**) (**B**)

Figure S4 HLA-A,B,C expression levels after infection of Calu-3 cells with SARS-CoV-2 strains non-VOC, Alpha, Beta, Gamma, and Delta, preliminary experiment. Calu-3 cells were infected with the different virus strains at MOI 0.01. Non-infected samples with identical treatment served as negative control. At indicated time points cells were analyzed. MFI: Mean Fluorescence Intensity. MFI of HLA-A,B,C-expression on the surface of **(A)** spike protein-positive (infected) and **(B)** spike protein-negative bystander Calu-3-cells. (n=1).

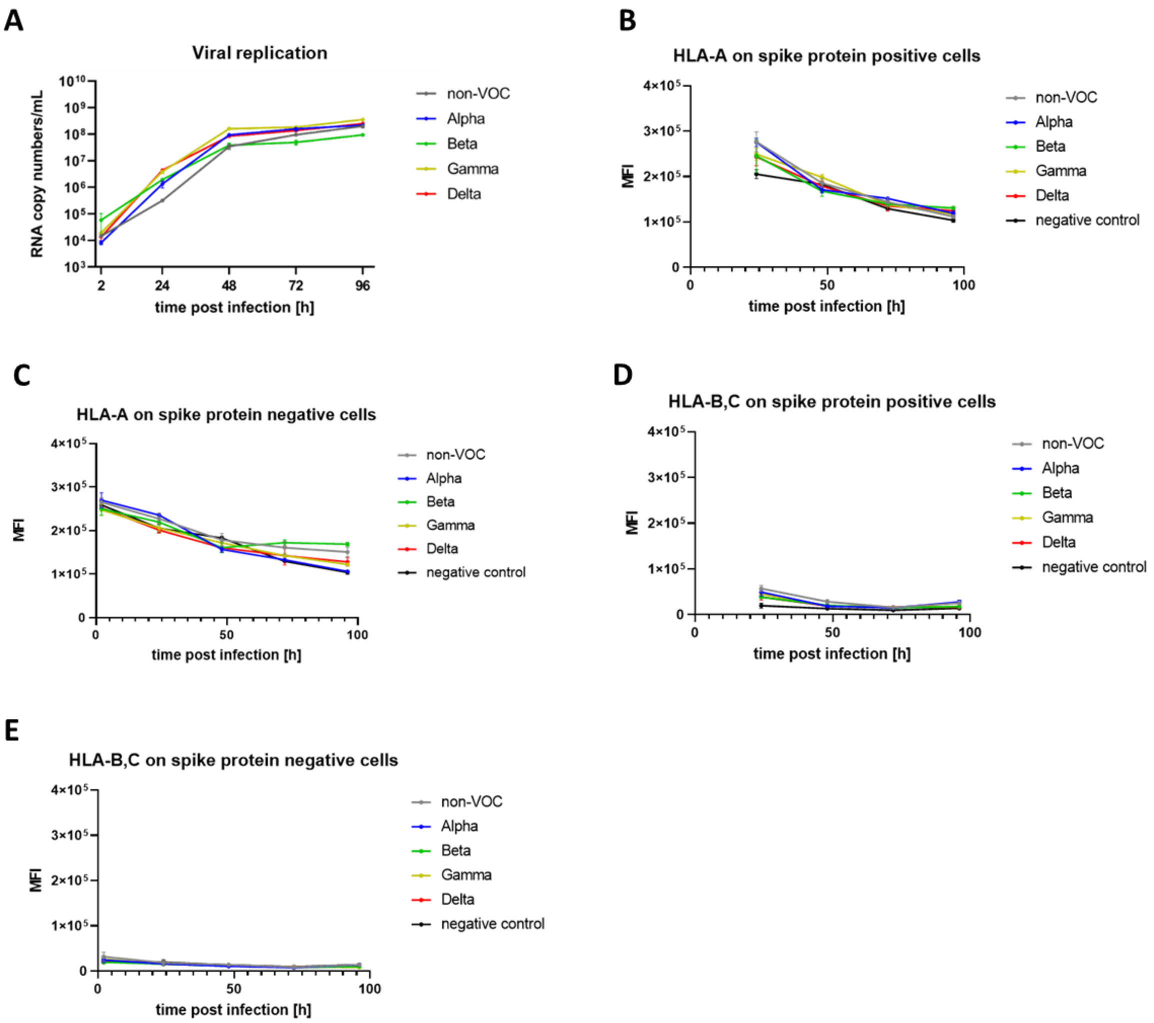

Figure S5: Time course of spike protein, HLA-A and HLA-B,C expression levels after infection of Caco-2 cells with SARS-CoV-2 strains non-VOC, Alpha, Beta, Gamma and Delta. Caco-2 cells were infected with the respective virus strains at MOI 0.01. Non-infected samples with identical treatment served as negative control. At indicated time points cells were analyzed in flow cytometry. MFI: Mean Fluorescence Intensity. **(A**) SARS-CoV-2 specific RT-qPCR analysis of supernatant to track viral replication. **(B)** MFI of HLA-A-expression on the surface of spike protein positive Caco-2 cells. **(C)** MFI of HLA-A-expression on the surface of spike protein-negative Caco-2 cells. **(D)** MFI of HLA-B/C expression on the surface of spike protein-positive Caco-2 cells **(E)** MFI of HLA-B/C expression on the surface of spike protein-negative Caco-2 cells. Error bars represent standard deviation (n=3).

Supplementary Table S1. HLA-1 staining in lung tissue samples of 3 SARS-CoV2 deceased patients. -/+: negative to very weak, +: weak, ++: moderate, +++: strong.

Tissue sample	Pneumocytes	Endothelial cells	Macrophages	Lymphocytes	Comments
**Antibody HLA class I heavy chain**					
**Lung control**	-	+	-/+	++	
Sample #1	+++	++	++	++	
Sample #2	+++	++	++	++	
Sample #3	+++	+	++	++	
**Antibody ß2-microglobulin**					
**Lung control**	-	-/+	+	-/+	
Sample #1	++	+	++	-/+	
Sample #2	-/+	-	+	-/+	
Sample #3	+	+	++	+	
